# Similarity-driven motion-resolved reconstruction for ferumoxytol-enhanced whole-heart MRI in congenital heart disease

**DOI:** 10.1371/journal.pone.0304612

**Published:** 2024-06-13

**Authors:** Ludovica Romanin, Bastien Milani, Christopher W. Roy, Jérôme Yerly, Aurélien Bustin, Salim Si-mohamed, Milan Prsa, Tobias Rutz, Estelle Tenisch, Juerg Schwitter, Matthias Stuber, Davide Piccini

**Affiliations:** 1 Department of Radiology, Lausanne University Hospital and University of Lausanne, Lausanne, Switzerland; 2 Advanced Clinical Imaging Technology, Siemens Healthineers International AG, Lausanne, Switzerland; 3 Center for Biomedical Imaging (CIBM), Lausanne, Switzerland; 4 IHU LIRYC, Electrophysiology and Heart Modeling Institute, Université de Bordeaux – INSERM U1045, Pessac, France; 5 Department of Cardiovascular Imaging, Hôpital Cardiologique du Haut-Lévêque, CHU de Bordeaux, Pessac, France; 6 University Lyon, INSA-Lyon, University Claude Bernard Lyon 1, UJM-Saint Etienne, CNRS, Inserm, CREATIS UMR 5220, U1206, Villeurbanne, France; 7 Department of Radiology, Louis Pradel Hospital, Hospices Civils de Lyon, Bron, France; 8 Division of Pediatric Cardiology, Woman-Mother-Child Department, Lausanne University Hospital and University of Lausanne, Lausanne, Switzerland; 9 Division of Cardiology, Cardiovascular Department, Lausanne University Hospital, Lausanne, Switzerland; 10 Faculty of Biology&Medicine, University of Lausanne, UniL, Lausanne, Switzerland; 11 Cardiac MR Center of the University Hospital Lausanne, Lausanne, Switzerland; Khalifa University, UNITED ARAB EMIRATES

## Abstract

A similarity-driven multi-dimensional binning algorithm (SIMBA) reconstruction of free-running cardiac magnetic resonance imaging data was previously proposed. While very efficient and fast, the original SIMBA focused only on the reconstruction of a single motion-consistent cluster, discarding the remaining data acquired. However, the redundant data clustered by similarity may be exploited to further improve image quality. In this work, we propose a novel compressed sensing (CS) reconstruction that performs an effective regularization over the clustering dimension, thanks to the integration of inter-cluster motion compensation (XD-MC-SIMBA). This reconstruction was applied to free-running ferumoxytol-enhanced datasets from 24 patients with congenital heart disease, and compared to the original SIMBA, the same XD-MC-SIMBA reconstruction but without motion compensation (XD-SIMBA), and a 5D motion-resolved CS reconstruction using the free-running framework (FRF). The resulting images were compared in terms of lung-liver and blood-myocardium sharpness, blood-myocardium contrast ratio, and visible length and sharpness of the coronary arteries. Moreover, an automated image quality score (IQS) was assigned using a pretrained deep neural network. The lung-liver sharpness and blood-myocardium sharpness were significantly higher in XD-MC-SIMBA and FRF. Consistent with these findings, the IQS analysis revealed that image quality for XD-MC-SIMBA was improved in 18 of 24 cases, compared to SIMBA. We successfully tested the hypothesis that multiple motion-consistent SIMBA clusters can be exploited to improve the quality of ferumoxytol-enhanced cardiac MRI when inter-cluster motion-compensation is integrated as part of a CS reconstruction.

## Introduction

The use of whole-heart coronary magnetic resonance angiography (CMRA) has been investigated during the past decades as a non-invasive and radiation-free approach to the diagnosis of coronary artery disease [1] and to the assessment of anomalous coronary arteries [2–4]. Conventional whole-heart CMRA acquisitions require prolonged scan time and are therefore highly sensitive to physiological motion [5]. Respiratory and cardiac motion produces artifacts that adversely affect the image quality and prevent a correct anatomical characterization of the heart and its vasculature. Traditionally, respiratory artifacts are suppressed using navigator-gating [6] or breath-holding while the mainstay for cardiac motion suppression is ECG-triggering or gating [7].

Alternatively, ungated or untriggered acquisitions have shown to replace navigators by the extraction of self-navigation signals directly from the imaging data to retrospectively reconstruct cardiac and respiratory resolved images [8–16]. These free-running approaches have the potential to simplify cardiac MRI by deliberately shifting the motion management from the acquisition side to the reconstruction. All of these techniques are free-breathing and resolve the cardiac motion retrospectively, either by using ECG signals [10, 11, 13] or by extracting a cardiac self-navigation signal, corresponding to the change of the ventricular blood volume [8, 9, 12, 14–16].

XD-GRASP [17] was proposed as a multi-dimensional motion-resolved compressed sensing (CS) technique in which information among similar motion states is shared in new dynamic sparse temporal dimensions. Building on this method, a fully automated framework for both self-gated cardiac and respiratory motion-resolved 5D whole-heart MRI was previously published [14]. As this approach separates the data into motion-consistent states through phyisiological binning, the reconstruction of 5D (3D spatial + cardiac motion + respiratory motion) images makes use of CS approaches to reduce undersampling artefacts and recover high-quality images [18]. 5D reconstructions rely on the explicit extraction of self-gating signals, and commonly assume a certain regularity of respiratory and cardiac motion over time, within predefined frequency ranges. A similarity-driven multi-dimensional binning algorithm (SIMBA) has been proposed as a fast (sub-minute) method for the reconstruction of free-running data [19]. SIMBA implicitly exploits the periodicity of physiological signals, captured as spatial similarities in the numerous k-space profiles of the free-running acquisition, to cluster motion-consistent data together. Combined with the injection of ferumoxytol [20], it was demonstrated that SIMBA results in diagnostic whole-heart volumes, which provide information about both the heart and coronary arteries with a quality similar to that obtained with the 5D CS reconstruction [19]. However, in classical SIMBA, only one among multiple motion-consistent clusters is selected for reconstruction, using a simple direct non-uniform fast Fourier transform (NUFFT), resulting in a large percentage of the data being discarded.

We posit that the additional information contained in these discarded clusters can be more exhaustively exploited for improved image quality and more efficient use of the abundant data collected during a free-running acquisition. Moreover, we demonstrate that with the addition of deformation fields in the reconstruction the data does not have to be in a sequential physiological order to be able to perform an effective regularization in CS.

Therefore, the goal of this work is to extend SIMBA with redundant information being shared in the clustering dimension. In the pursuit of this, we test two hypotheses: First, that the SIMBA clusters can be considered as a new dynamic dimension as part of a CS reconstruction approach that exploits the spatial redundancy of the anatomical information among the clusters. Second, that since it is not possible to predict what specific physiological phases the SIMBA algorithm returns in its most populated clusters, the sparsity of the clustering dimension will be heavily patient-dependent and often suboptimal (e.g., with systolic clusters close to diastolic clusters). By incorporating inter-cluster motion compensation into the CS reconstruction, large and unpredictable anatomical differences can be compensated, without compromising image quality.

These hypotheses were tested in a cohort of patients with congenital heart disease (CHD) after ferumoxytol-enhanced free-running 3D radial acquisitions using image quality metrics of the heart and the coronary arterial system as endpoints.

## Methods

### Ferumoxytol-enhanced CMR acquisitions

Twenty-four patients with CHD (age 25±15 years, range 2–60 years; 18 male; weight 64±24 kg, range 12.6–104 kg) with a clinical indication for ferumoxytol-enhanced cardiac MRI were included in this IRB-approved retrospective study (Commission cantonale d’éthique de la recherche sur l’être humain, CER-VD, approval number 2022–01521). Data was accessed for research purposes between 20.10.2022 and 08.12.2022, together with the patients’ demographics and characteristics. Each study participant or their legal guardian provided IRB-approved written informed consent. The datasets were obtained from consecutively recruited patients who were scanned with an identical imaging protocol.

In vivo acquisitions were performed on a 1.5T clinical MRI scanner (MAGNETOM Sola, Siemens Healthcare, Erlangen, Germany). The free-running research application sequence used in this study is a gradient-echo sequence without fat saturation pre-pulses, which has been previously published [21]. K-Space data were continuously sampled using a 3D golden angle kooshball phyllotaxis trajectory [22], interleaved with the frequent acquisition of superior-inferior (SI) readouts, which are commonly used for the extraction of self-gating cardiac and respiratory signals [14]. The acquisition protocol consists of 5’749 radial interleaves and 22 readouts/interleave. The main sequence parameters were as follows: radio frequency excitation angle of 15° with an axial slab-selective sinc pulse, resolution of 1.15–1.35 mm^3^, FOV of 220–260 mm^3^, TE/TR of 1.64/2.84 ms, and readout bandwidth of 1002 Hz/pixel. The SI readouts are played out with a frequency of 16Hz. The total scan time was 5:59 minutes.

All examinations were performed during free-breathing after administration of 2 to 4 mg/kg of ferumoxytol (Feraheme, AMAG Pharmaceuticals, Waltham, Massachusetts, USA) [20] as a slow-infusion over 15 minutes.

### Image reconstruction

#### Similarity-driven multidimensional binning algorithm (SIMBA)

As a common starting point to all reconstructions, we apply SIMBA as described in the original publication [19], to have a fast binning of the data into different motion-consistent clusters, without explicitly extracting or resolving the whole range of physiological motion. In summary, this technique consists of two main steps: i) the acquired SI projections are concatenated into a single 2D matrix that undergoes a dimensionality reduction by applying a principal component analysis (PCA), ii) only the real part of the data projected in this PCA space is clustered using k-means into 10 to 14 disjoint clusters. As described by Heerfordt et al. [19], this range was prospectively chosen to have approximately 12000–15000 readouts (acceleration factor of 4 to 5), adequate for the reconstruction of a 3D whole-heart volume in the most populated cluster. The final optimal number of clusters, within this search range, is then determined by using an automated search procedure which aims at selecting a number of clusters *k* for which the average distance between the cluster’s centroid *μ*_*k*_ and the data points $\hat{s}$ of the most populated cluster *C* is minimized:${\text{argmin}}_{k\;\in \;\left\{10,\ldots ,14\right\}}\frac{1}{\left|C\right|}{\sum_{\hat{s}\;\in \;C}\left\|\mu_{k}-\hat{s}\right\|}_{2}$.

Each of the extracted clusters of data is reconstructed using a 3D gridded reconstruction for non-Cartesian acquisitions, consisting of a density compensation, NUFFT [23], and application of the coil sensitivities, which were estimated from the pre-scan calibration data using a modified version of the method presented by Pruessmann et al. [24] and presented in Milani et al. [25].

The original SIMBA method only considers the most populated cluster of data for image reconstruction. It was shown that these images have the best image quality relative to other clusters and generally target a diastolic and end-expiratory resting phase [19].

#### Motion-resolved reconstruction (XD-SIMBA)

To make better use of the multiple SIMBA clusters, we optimized the original SIMBA algorithm by selecting not just one but four of the motion-consistent clusters for reconstruction. This was chosen empirically by targeting the use of approximately half of the acquired data, which corresponds to 4–5 clusters having 12000–15000 readouts in the largest clusters. To account for the variability in the number of readouts per cluster across subjects, we took a minimum of four clusters as a tradeoff between potential image quality and computation time. The different clusters are representative of the same anatomy in different states, so we can make use of a CS concept introduced in the original XD-GRASP publication [17] and perform a motion-resolved reconstruction in which the 3D volumes reconstructed from the four most populated SIMBA clusters become a new dynamic dimension (XD-SIMBA). The reconstruction problem can be formulated as:

$$\hat{x}=argmin_{x}\;\;{\sum_{i=1}^{K}\left\|F^{\left(i\right)}Cx^{\left(i\right)}-y^{\left(i\right)}\right\|}_{2}^{2}+\lambda {\sum_{i=1}^{K}\left\|x^{\left(i\right)}-x^{\left(i-1\right)}\right\|}_{1}$$ (1)

where *F* is the NUFFT, *C* the coil sensitivities for all coils applied as a matrix block $\left({\left[C_{1}\ldots C_{N_{coils}}\right]}^{\text{T}}\right)$ in the same way for all clusters, *x*^(*i*)^ the static 3D image reconstructed from the cluster *i* where cyclical motion was considered by setting *x*^(0)^ = *x*^(*K*)^, *y*^(*i*)^ the k-space data in the cluster *i*, and *K* the number of SIMBA clusters chosen (K = 4). ${\left\|\cdot \right\|}_{2}$ is the L2-norm and ${\left\|\cdot \right\|}_{1}$ is the L1-norm. The total variation regularization parameter *λ* was experimentally optimized (see S1 Fig) and was set to 0.3. This optimization problem was solved via the alternating direction method of multipliers (ADMM), and the alternating minimization problem using the conjugate gradient (CG) method. From the resultant four datasets, the XD-SIMBA image originating from the most populated cluster was then used for analyses.

#### 3D motion-resolved reconstruction with inter-cluster motion compensation (XD-MC-SIMBA)

As SIMBA clustering does not necessarily lead to the extraction of motion states that follow each other in a physiological sense, we compensate for potentially large deformations by estimating 3D non-rigid deformation fields between pairs of gridded images from adjacent clusters using NiftyReg [26]. Motion compensation can be achieved by integrating the deformation fields inside the CS reconstruction framework [27–29]. Similarly, here the estimated 3D non-rigid deformation fields are incorporated into the reconstruction (XD-MC-SIMBA) by reformulating the problem in Eq (1) as:

$$\hat{x}=\;\underset{x}{\text{argmin}}{\sum_{i=1}^{\text{K}}\left\|F^{\left(i\right)}Cx^{\left(i\right)}-y^{\left(i\right)}\right\|}_{2}^{2}+\lambda {\sum_{i=1}^{\text{K}}\left\|T_{u}^{\left(i\right)}x^{\left(i\right)}-x^{\left(i-1\right)}\right\|}_{1}$$ (2)

Where $T_{u}^{\left(i\right)}$ is the non-rigid image deformation operator that deforms *x*^(*i*)^ in order to match *x*^(*i*−1)^, where cyclical motion was considered by setting *x*^(0)^ = *x*^(*K*)^. Estimation of the deformation fields was obtained by optimizing an objective function based on the Normalized Mutual Information between *x*^(*i*)^ and *x*^(*i*−1)^, and the Niftyreg program was applied with the default parameters except for the maximal number of iterations raised to 300. To evaluate the contribution of such motion registration to the final image quality, we apply the same regularization factor as for XD-SIMBA, meaning a *λ* of 0.3. For comparison with SIMBA and XD-SIMBA, the XD-MC-SIMBA image was the one in the same motion state as the most populated SIMBA cluster.

For more details about the formulation and implementation of the image reconstruction problem please refer to the work of Milani et al. [25].

**Fig 1:**
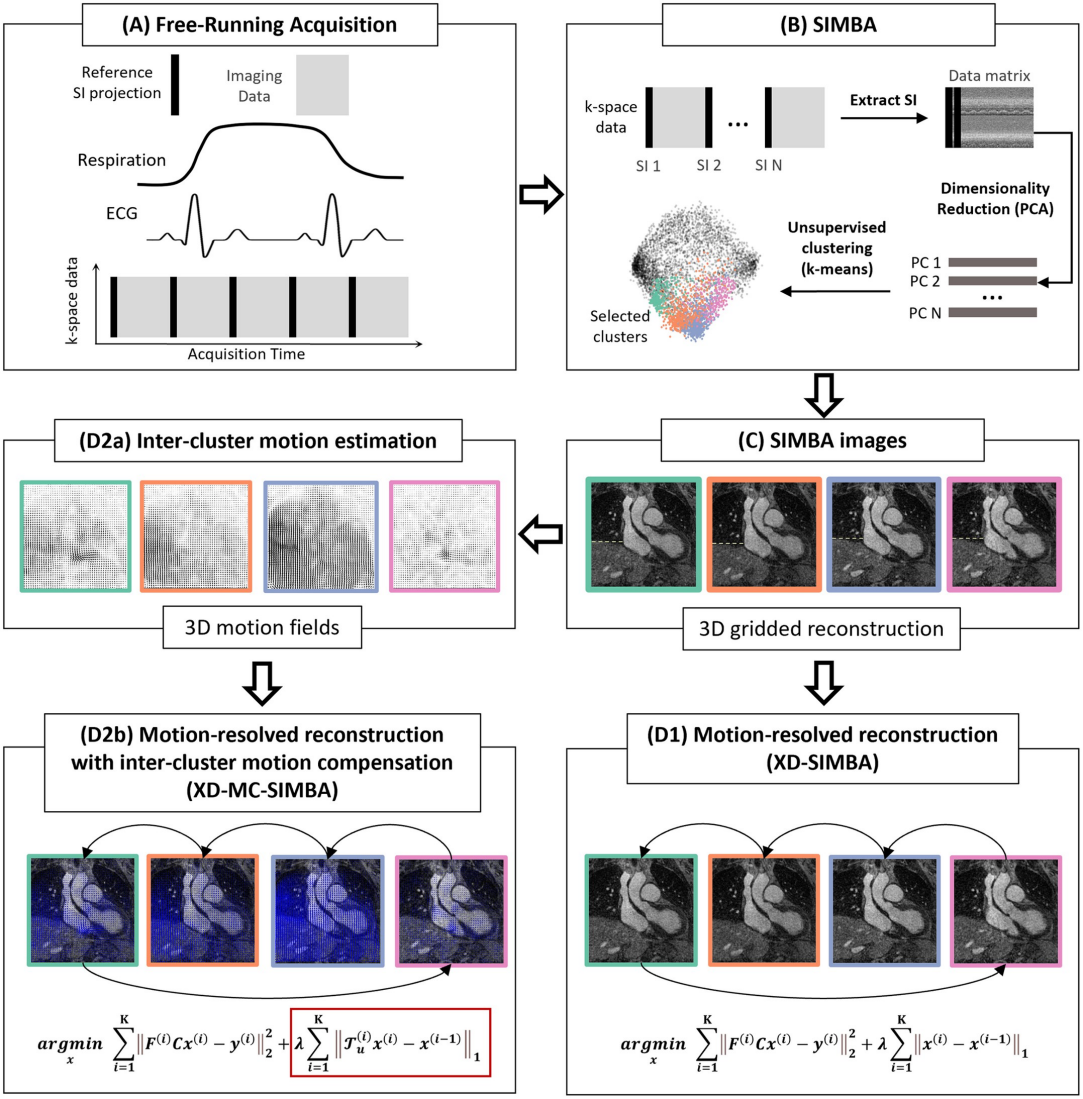
Summary of the main steps involved in the image reconstruction. Starting from a free-running acquisition (A), the reference SI projections are concatenated to obtain a matrix that is used as input to SIMBA (B). After applying SIMBA, the resulting binning consists of a set of disjoint clusters. As a first common step, the four most populated clusters are selected and reconstructed using a non-uniform 3D gridded reconstruction (C). The original SIMBA image consists of taking the non-uniform 3D gridded reconstruction of the most populated cluster. (D1) A motion-resolved reconstruction (XD-SIMBA) is obtained by performing a compressed-sensing reconstruction and regularization over the clustering dimension. (D2a) Additionally, we integrate a non-rigid motion estimation in the iterative reconstruction framework (D2b) to regularize over co-registered clusters and obtain a motion-resolved reconstruction with inter-cluster motion compensation (XD-MC-SIMBA).

All 24 datasets were reconstructed with the three reconstructions (SIMBA, XD-SIMBA, XD-MC-SIMBA), as visually summarized in Fig 1. All reconstructions (both 3D gridded and motion-resolved) were performed with a GeForce RTX 3090TI GPU with 24 GB of VRAM, while the other operations (SIMBA clustering and image registration) were carried out on CPU. The ADMM iterations were set to 40, while the CG iterations were set to 3.

#### Free-running framework (FRF) 5D image reconstruction

In addition, the same 24 datasets were reconstructed using the published free-running framework (FRF) for 5D image reconstruction of ferumoxytol-enhanced datasets [21], a CS reconstruction that exploits sparsity along both the cardiac and the respiratory dimensions, obtained by explicitly extracting self-gating cardiac and respiratory motion signals. For the FRF, the ADMM iterations were set to 10, while the CG iterations to 4. Total variation regularization weights were the same for both cardiac and respiratory dimensions and set to 0.01. This reconstruction is compared to all other reconstructions.

### Image quality analysis

To objectively compare the quality of the four different reconstructions, the following metrics were calculated. The contrast ratio between blood and myocardium was assessed by computing the ratio between the difference in signal intensity of blood and myocardium divided by the myocardial signal intensity. The sharpness of the lung-liver and blood-myocardium interfaces were quantified by fitting parametrized sigmoid functions to the tissue interfaces, with the slope parameter representing the sharpness value [30]. The total visible length and sharpness of the first 4 cm of the right coronary artery (RCA) and the combined left main (LM) and left anterior descending coronary artery (LAD) were quantified using the Soap-Bubble tool [31]. Furthermore, image quality scores (IQS) were assigned to each whole-heart 3D volume by using a previously published deep learning-based approach for image quality assessment [32]. This algorithm was trained to assign grades according to the following scale: 0, non-diagnostic; 1, limited diagnostic value; 2, image of diagnostic value; 3, good diagnostic value; 4, excellent diagnostic value. By using this automated approach, the IQS assignment is blinded to the reconstruction type and can identify differences in image quality when applying different reconstruction methods [32] (S1 Appendix).

Analysis of sharpness, contrast ratio, and coronary artery metrics were only performed on the images resulting from the most populated cluster [19] and from a phase from the 5D FRF reconstruction that mostly resembled the state depicted in the SIMBA images. We assigned IQS to the selected FRF image, and final SIMBA, XD-SIMBA and XD-MC-SIMBA images, from the most populated cluster. We reported the relative % difference in IQS grades between pairs of reconstructions.

The average optimal number of SIMBA clusters across subjects was recorded, and the percentage of the acquired data used for each SIMBA reconstruction was reported together with the undersampling factor R. In addition, the reconstruction times were measured.

Statistical analyses of all quantitative metrics were performed using one-way analysis of variance (ANOVA) with Bonferroni correction to account for multiple comparisons [33]. Statistical significance was defined by two-sided paired sample t-tests with p<0.0125. All image reconstructions and analyses were performed using MATLAB (ver. R2021, The MathWorks, Natick, Massachusetts, USA).

## Results

The SIMBA clustering resulted in 13 clusters on average per patient (see S2 Fig on the size and shape of the clusters). Among the four most populated clusters selected for the reconstruction, the first contained 12.2±1.9% of the acquired data, the second 10.5±1.4%, the third 9.6±1.2%, and the fourth 8.9±1.0%. In terms of undersampling factors, the first cluster was undersampled by a factor of R = 4, the second by R = 4.5, the third by R = 5, and the fourth by R = 5.4. The average computation time for the different reconstructions (including also clustering, binning and estimation of the deformation fields, when applicable) were: 1.8 min ± 25 sec (SIMBA), 2.4 hours ± 24 min (XD-SIMBA), 3.2 ± 1 hours (XD-MC-SIMBA), and 7.5 ± 1 hours (FRF).

**Fig 2:**
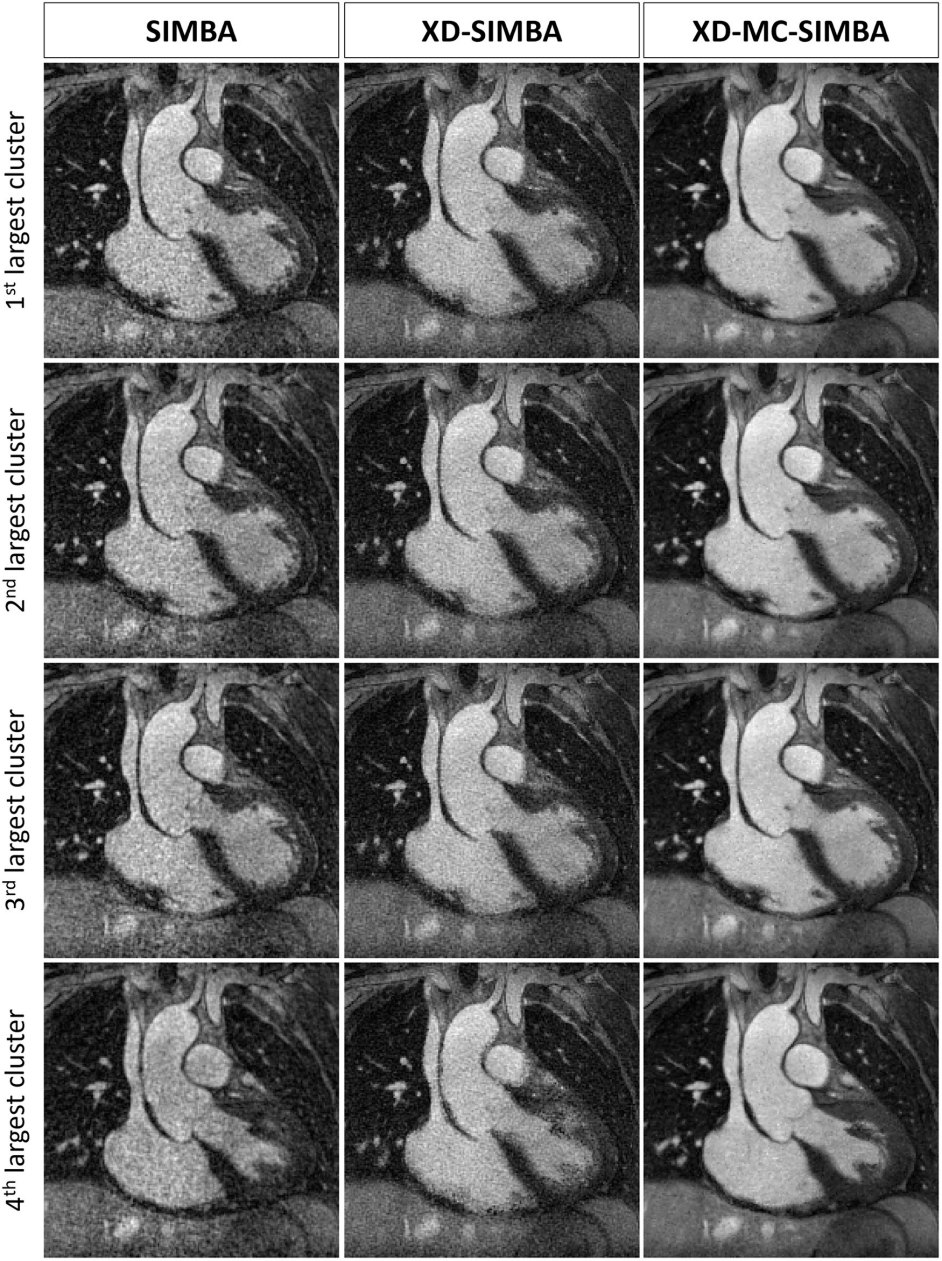
Example of the different images reconstructed from the four selected SIMBA clusters using a simple gridded reconstruction (SIMBA), a CS reconstruction for which the SIMBA clustering is a dimension of sparsity (XD-SIMBA), and the same CS reconstruction extended with inter-cluster motion compensation (XD-MC-SIMBA). The data shown are from a patient with tetralogy of Fallot (M, 36 years). The indication for the ferumoxytol-enhanced scan was a transannular patch repair.

As expected, the four selected SIMBA clusters can depict physiological states of the heart that are very similar or very different, e.g. diastole for the first three clusters and systole for the fourth cluster (Fig 2). Hence during the CS reconstruction, the images from the clusters adjacent to this fourth one, i.e. the third and the first one when assuming circular motion, are negatively affected by this large uncompensated deformation. Indeed, we observe that the simple CS reconstruction (XD-SIMBA) results in blurrier images, compared to the gridded images (SIMBA), especially in the left ventricular region, where most of the deformations of the myocardium are observed. This is particularly visible for the fourth image, for which it is no longer possible to distinguish the border between the blood-pool and the myocardium. However, when compensating for inter-cluster motion with the integration of deformation fields inside the CS reconstruction (XD-MC-SIMBA), the blurring is no longer observed, but instead we have very sharp images for all clusters, even compared to the original SIMBA reconstructions (see S3 Fig about the performance of image registration).

**Fig 3:**
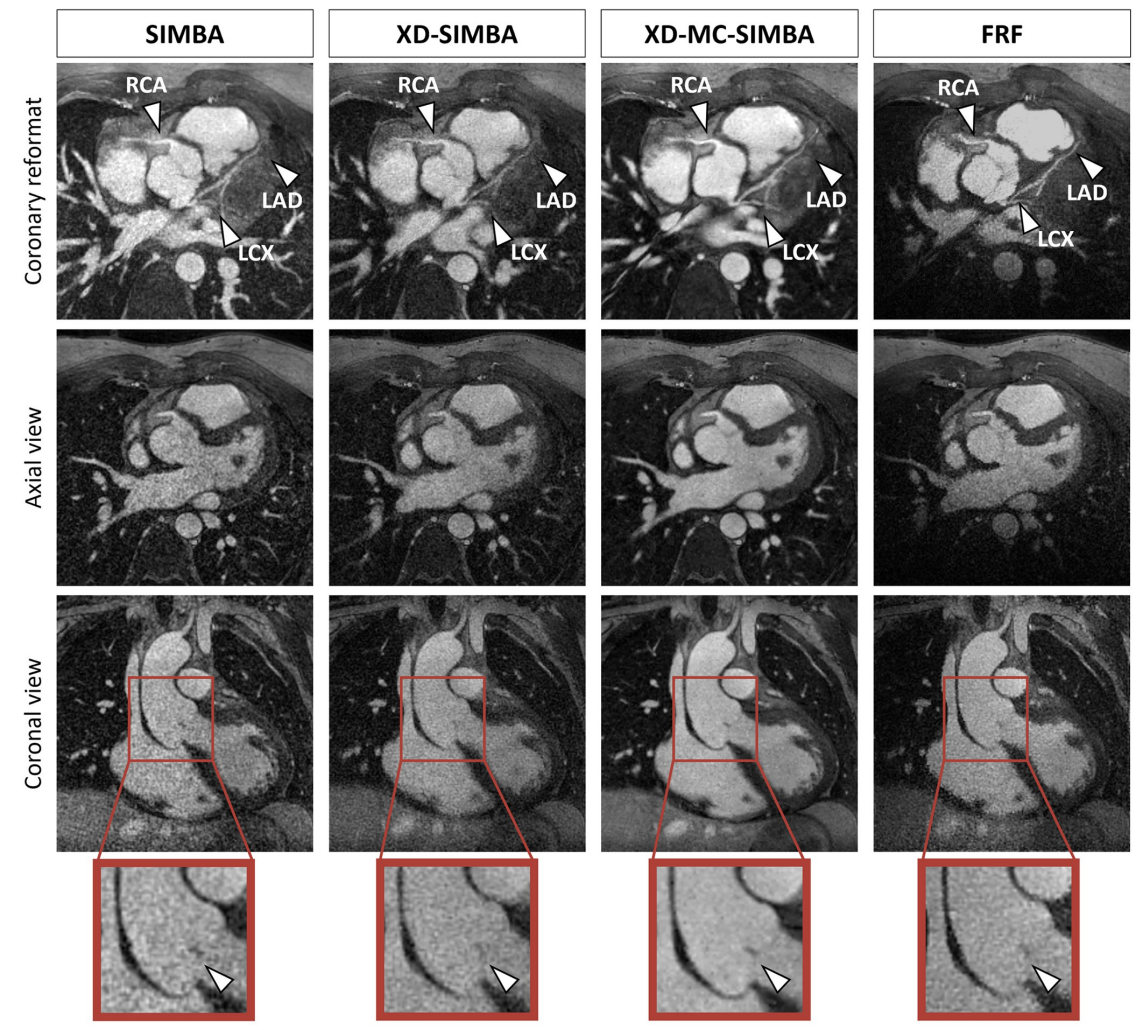
Example (also shown in Fig 2) of a patient after repair of tetralogy of Fallot and zoomed-in view of the aortic valve. The coaptation area of the aortic leaflet (arrow) is more clearly visible in XD-MC-SIMBA and FRF. In this example, XD-MC-SIMBA has a much higher assigned image quality score, even compared to FRF. When looking at the coronary reformat, the distal portions of the left anterior descending (LAD) and left circumflex (LCX) coronary arteries are only depicted in XD-MC-SIMBA and FRF, and the proximal right coronary artery (RCA) is also better delineated in XD-MC-SIMBA, even compared to FRF. Abbreviations: RCA, right coronary artery; LM, left main coronary artery; LAD, left anterior descending.

Zooming into smaller anatomical structures, we observe how XD-MC-SIMBA offers a much improved visualization of the coronary arteries and aortic valve, compared to both SIMBA and XD-SIMBA (Fig 3). This case shows how XD-SIMBA, because of uncompensated and possibly large motion between neighboring clusters, may not be sufficient to recover anatomical detail and may even lead to blurring of the coronary vessels. When comparing these reconstructions to that of 5D FRF, we see how the FRF image has higher image quality compared to both SIMBA and XD-SIMBA, with the depiction of the coronary vessels or the aortic valve being very similar to that from XD-MC-SIMBA.

**Fig 4:**
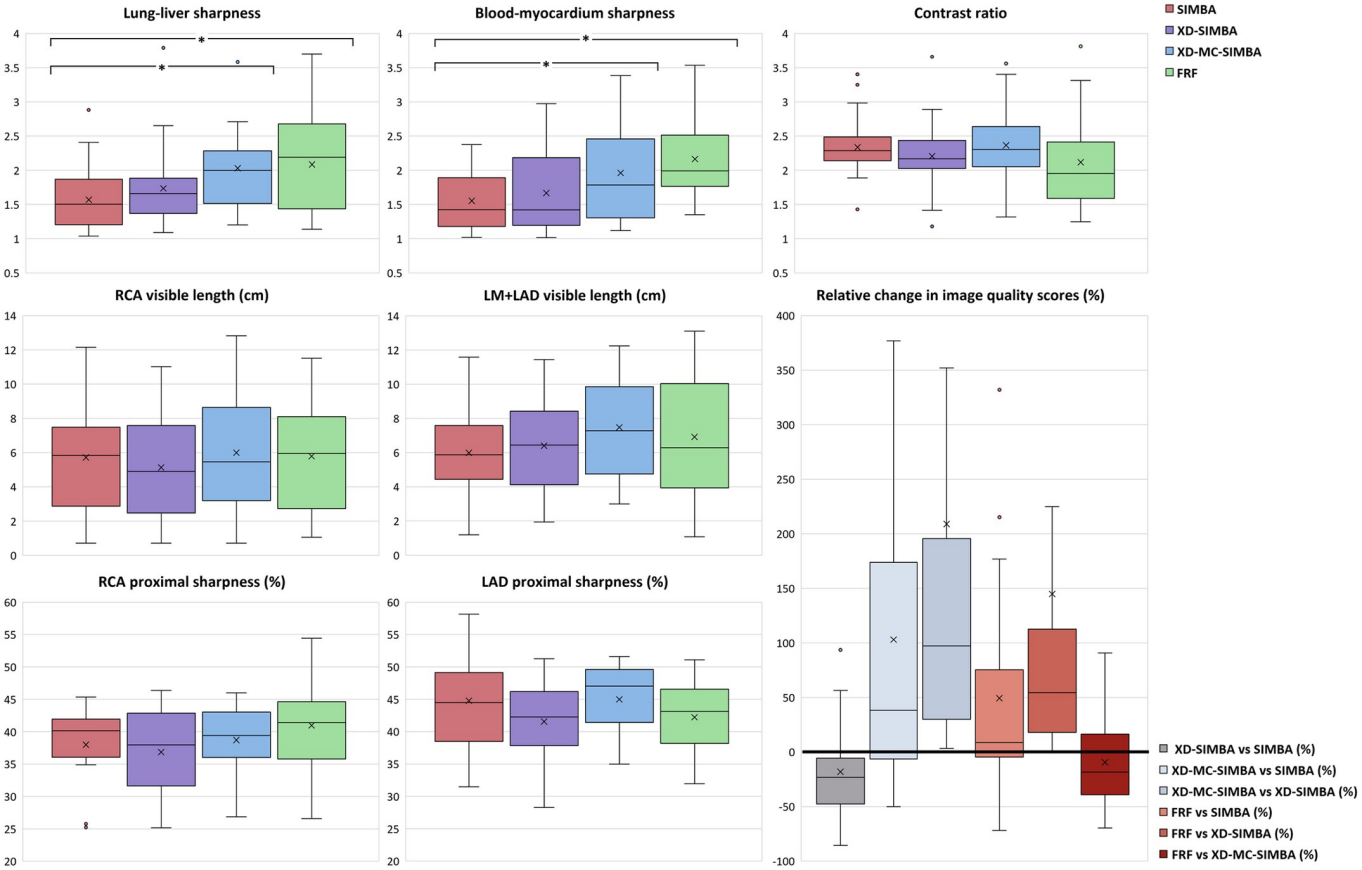
Quantitative image analysis metrics for the 3D gridded reconstruction (SIMBA) in red, the motion-resolved reconstruction (XD-SIMBA) in purple, the motion-resolved reconstruction with inter-cluster motion compensation (XD-MC-SIMBA) in blue, and a resting phase from the 5D free-running reconstruction (FRF) in green. Analysis of the LAD and RCA coronary arteries comprises the total visible vessel length, and the sharpness of the first proximal 4 cm. All results are shown using box plots, where the “x” indicates the mean values and the solid line the median values. Differences that are statistically significant are indicated by an asterisk (*) for p<0.0125. When looking at the relative percentage changes in image quality scores (IQS), overall XD-SIMBA results in a decrease in image quality score compared to SIMBA. Conversely, XD-MC-SIMBA improves the image quality compared to both SIMBA and XD-SIMBA. For FRF, there is also an improvement compared to both SIMBA and XD-SIMBA, but a slight worsening in score compared to XD-MC-SIMBA. Abbreviations: LM+LAD, left main+left anterior descending; RCA, right coronary artery.

The lung-liver sharpness is significantly higher in XD-MC-SIMBA compared to SIMBA (p = 0.0004), but not compared to XD-SIMBA (p = 0.02). For FRF, it is significantly higher compared to SIMBA (p = 0.003), but not compared to XD-SIMBA (p = 0.09) or XD-MC-SIMBA (p = 0.86). The blood-myocardium sharpness is also significantly higher in XD-MC-SIMBA compared to SIMBA (p = 0.0002), but neither compared to XD-SIMBA (p = 0.03) nor compared to FRF (p = 0.23). The blood-myocardium contrast ratio does not show significant differences among the methods (Fig 4; Table 1).

**Table 1:** Summary of all metrics values (mean standard deviation) for the different reconstructions, with the corresponding statistical analysis (p-values) using one-way analysis of variance (ANOVA).

METRICS	RECONSTRUCTION ALGORITHM				p-value
	SIMBA	XD-SIMBA	XD-MC-SIMBA	FRF	
Lung-liver sharpness	1.54±0.45	1.77±0.57	2.05±0.63	2.08±0.70	0.008
Blood-myocardium sharpness	1.60±0.40	1.70±0.56	1.96±0.65	2.17±0.60	0.003
Contrast ratio (blood to myocardium)	2.35±0.45	2.21±0.50	2.40±0.52	2.15±0.67	0.416
RCA visible length (cm)	6.14±3.31	5.39±2.99	6.31±3.37	6.25±2.85	0.771
RCA proximal sharpness (%)	38.8±4.93	38.0±6.12	39.49±5.05	42.2±7.54	0.445
LM+LAD visible length (cm)	6.12±2.45	6.64±2.73	7.79±3.03	7.42±3.58	0.265
LAD proximal sharpness (%)	45.1±7.22	41.4±6.03	44.5±5.52	42.9±5.46	0.337

Analysis of the coronary arteries (Fig 4) indicates a trend for a higher average total visible vessel length in XD-MC-SIMBA, for LM+LAD and RCA compared to SIMBA and XD-SIMBA, although this was not found to be statistically significant. The vessel lengths are the highest for FRF. Computation of the sharpness for the first proximal 4 cm of the RCA were higher for XD-MC-SIMBA in the first 4 cm compared to SIMBA and XD-SIMBA, while not statistically significant. FRF gives the highest RCA sharpness. Sharpness measures of the LAD are very similar between SIMBA and XD-MC-SIMBA while lower for XD-SIMBA and FRF, even though not statistically significant. Only in 2 and 3 cases the left and right coronary systems respectively were not visible, but this was the case for all SIMBA reconstructions as the resolution was not high enough to see such small vessels. In 2 additional cases, FRF resulted in lower visibility of the LAD. In 1 case the RCA was only visible with FRF (Table 2).

**Table 2:** Count of visible ostia, visible proximal and distal portions of the RCA and LAD for all analyzed cases. Reported scores are from all 24 cases.

METRICS	RECONSTRUCTION ALGORITHM			
	SIMBA	XD-SIMBA	XD-MC-SIMBA	FRF
Visible RCA ostium	21	21	21	22
Visible RCA proximal portion	18	17	18	20
Visible RCA distal portion	4	4	7	7
Visible LM ostium	22	22	22	22
Visible LAD proximal portion	22	22	22	20
Visible LAD distal portion	6	7	8	10

For the SIMBA reconstructions, both RCA and LM coronary ostia were visible in the same cases, while SIMBA and XD-SIMBA show lower counts of visible proximal and distal portions of the RCA and LAD coronary arteries, compared to SIMBA. For the FRF reconstruction, an additional RCA vessel can be observed, compared to SIMBA, while there two less visible LAD vessels.

The IQS comparison (Fig 4) demonstrates how XD-SIMBA resulted in a decrease in IQS (-18±40%) when compared to SIMBA for 19 out of 24 cases. Conversely, XD-MC-SIMBA led to an improved image quality (+103±154%) in 18 out of 24 cases. For FRF there was an increase in IQS compared to both SIMBA (+49±88%) and XD-SIMBA (145±355%), but a slight decrease compared to XD-MC-SIMBA (-9±42%).

**Fig 5:**
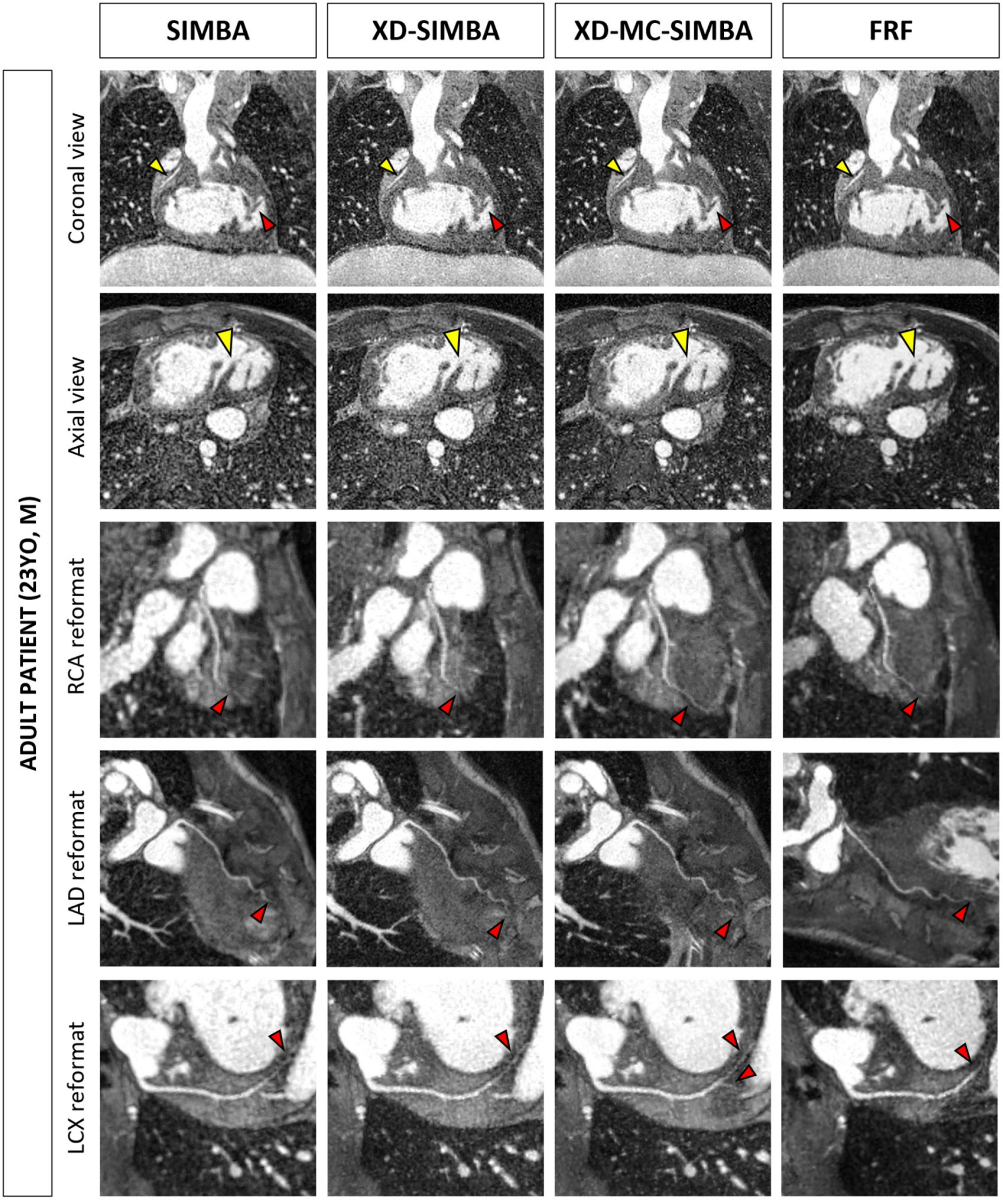
23-year-old male patient post Fontan procedure, with a right atrial isomerism. In the coronal view, the RCA is clearly visible (yellow arrow), and sharper for the XD-SIMBA and XD-MC-SIMBA reconstructions. Moreover, the papillary muscles (red arrow) are better delineated for XD-MC-SIMBA. This is observed also in the axial view (yellow arrow). When looking at the coronary reformats, the more distal segments of the RCA, LAD and LCX can be visualized in the XD-MC-SIMBA reconstruction (red arrows). Also for FRF there is a very good depiction of the cardiac anatomy, while only the coronary vessels seem less visible, compared to XD-MC-SIMBA. Abbreviations: RCA, right coronary artery; LAD, left anterior descending; LCX: left circumflex.

Focusing on the depiction of specific anatomical features, such as the papillary muscles and coronary vessels, XD-MC-SIMBA results in sharper images (Fig 5). Moreover, the traceable length of both left and right coronary vessels increases, making even more distal portions of the coronaries visible using to XD-MC-SIMBA. The FRF image does not provide equally high vessel conspicuity and sharpness as XD-MC-SIMBA (RCA sharpness: XD-MC-SIMBA 42.8% vs. FRF 40.4%).

**Fig 6:**
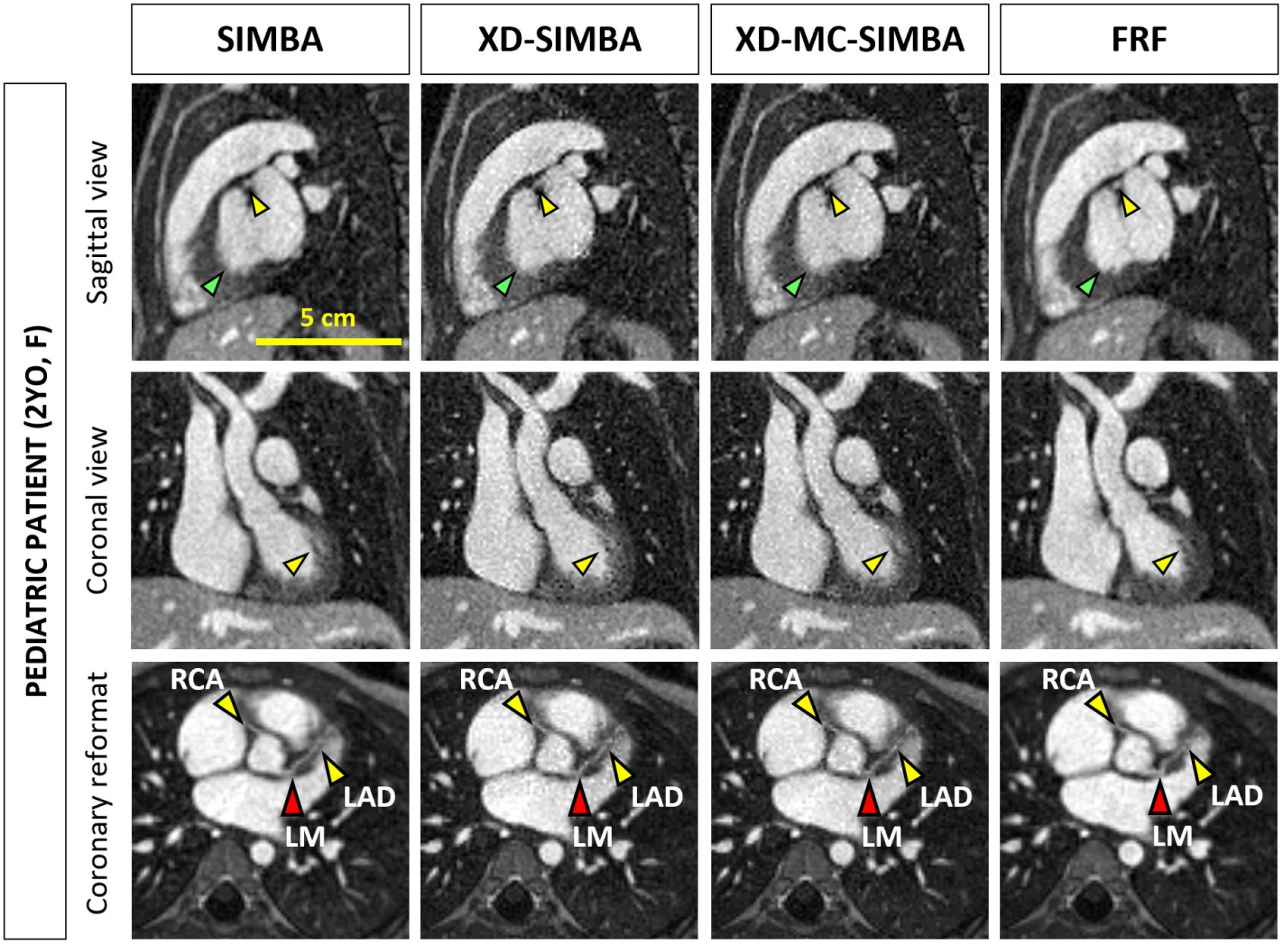
2-year-old female patient with Kawasaki disease. The yellow bar (first image on the left in the sagittal view) indicates the scale after the zoom around the heart (heart diameter <10cm). In the axial view, the right atrial wall is sharper for XD-SIMBA and XD-MC-SIMBA, compared to SIMBA (yellow arrow), and so are the papillary muscles in the left ventricle (red arrow). In the sagittal view, the yellow arrow indicates the LAD, while the green arrow indicates the left ventricular wall, which is sharper in FRF. In the coronal views, the papillary muscles (yellow arrow) are sharper compared in XD-MC-SIMBA, compared to both SIMBA and XD-SIMBA. For FRF, the image quality is good in terms of delineation of big anatomical features (e.g. papillary muscles, septal wall), but small features such as the coronary vessels are blurrier. Abbreviations: RCA, right coronary artery; LM, left main coronary artery; LAD, left anterior descending.

In the case of pediatric patients, the anatomical structures are much smaller compared to those of the adults and the visualization of the coronary arteries is more challenging (Fig 6). Nonetheless, both XD-SIMBA and XD-MC-SIMBA allow a better visualization of both right and left coronary arteries, with longer traceable lengths, and improved vessel conspicuity, particularly for the more distal segments, even compared to FRF. However, the myocardium and papillary muscles are better visualized with FRF.

A summary of all image quality metrics for each figure showed in the paper can be found in the Supporting Information (S4 Fig).

## Discussion

The SIMBA reconstruction was proposed as an effective method to suppress adverse effects of respiratory and cardiac motion in 3D free-running MRI, without an explicit extraction of physiological signals. In this work, we developed a new reconstruction (XD-MC-SIMBA) that further exploits the inherent abundance of information from a free-running acquisition by using the SIMBA clustering as a new dimension of sparsity for CS reconstruction. Without the inter-cluster motion compensation, XD-SIMBA is not able to achieve good image quality, because the anatomical state depicted in each cluster cannot be predicted. This method was refined with non-rigid inter-cluster deformation fields to further promote sparsity without any constraint on the selection or reordering of the clusters and improve the image quality.

Moreover, the XD-SIMBA and XD-MC-SIMBA reconstructions exploit 41% of the acquired data compared to only 12% of the data for SIMBA.

In terms of image quality metrics, our results suggest improved sharpness and coronary visibility for XD-MC-SIMBA, together with higher assigned IQS, relative to SIMBA. Compared to the 5D free-running FRF reconstruction, we documented comparable image quality, and in some instances even improved visibility and sharpness of coronary vessels. Conversely, XD-SIMBA resulted in an overall inferior image quality. This result can be explained with two main contributors. On the one hand, the motion deformation between adjacent clusters can be significant (e.g., systolic clusters close to diastolic clusters), and thus performing a total variation regularization is ineffective without the addition of inter-cluster motion compensation. On the other hand, to fulfill the sparsity condition in XD-SIMBA, the choice and ordering of clusters become crucial elements for a successful image reconstruction. Moreover, because of the high variability of adjacent SIMBA clusters across subjects, keeping the same regularization factor may have no undesired effects for similar cluster images, but it may cause sever blur for very different images. The optimization of the regularization factor is one of the most critical steps in CS reconstructions and requires accurate tuning. Previous publications reported an empirical optimization based on a visual image quality comparison [17, 34], which was also done in this work. Moreover, in Feng et al. [17], a series of reconstructions with different regularization factors was reported, showing how extensive regularization produces compression artifacts while insufficient regularization fails to adequately remove the undersampling artifacts. The performance of XD-SIMBA was comparable to SIMBA in only a few cases, which means that depending on the physiological states selected with SIMBA and the clusters’ order, we may have to tune *λ* for each patient individually, and for pairs of images depending on their similarity. This greatly hinders the applicability of the technique. XD-MC-SIMBA minimizes differences in-between images from adjacent clusters, and hence the dependency on *λ* is reduced.

Similar to the work of Correia et al. [35] and of Bustin et al. [36] integrating an inter-bin, or inter-cluster in our case, non-rigid registration inside an iterative reconstruction framework reduced the appearance of motion artifacts. However, we did not correct for residual intra-bin (or intra-cluster) motion. With the current approach to the SIMBA clustering, we clustered data according to similarity by only putting a constraint on the maximal number of clusters, meaning that two cardiac phases that are anatomically similar (e.g., same ventricular size) may be grouped together, although small features (e.g., the valve cusps) may not be identical. By using self-navigation signals in combination with autofocusing [37, 38], we may also correct for intra-cluster motion prior to the CS reconstruction. Another solution would entail the integration of intra-cluster deformation fields into the iterative CS reconstruction.

Although motion-resolved compressed sensing reconstructions should minimize temporal blurring in the case of large inter-frame (inter-cluster in this case) motion, the addition of a temporal regularization supports de-noising. Consequently, the images may appear filtered, with the risk of having anatomical contours less defined. As described in other publications, the blurring in CS is due to this denoising property and can render images non-diagnostic for high undersampling factors combined with a wrong choice of regularization parameters [39]. As an alternative to CS, exploiting the inherent redundancy in the anatomical information along the clustering dimension by using a patch-based regularization may be an interesting option [36, 40–42]. Assuming that each 3D volume can be represented in terms of a redundant dictionary of 3D patches, the regularization could be reformulated to include a self-similarity matrix built on these extracted patches. Thanks to this sparser image representation, we could extend our algorithm to consider also highly undersampled images (i.e. reconstructed from very small clusters of data) and increase even further our data efficiency, without introducing artifacts [43].

Having a motion-resolved CS reconstruction with inter-cluster motion compensation considerably increased the computation time, going from a few minutes of reconstruction to well above three hours. The goal of this work was not to improve the performance in terms of computation time, but to push the limits of the reconstruction framework by using the redundancy of information shared among clusters and obtain the best possible image quality. Strategies to decrease the total computation time are needed if we want to allow better clinical translation of our technique. Reducing the number of ADMM iterations, performing a coil compression, and optimizing the code via parallel processing or conversion of the code to a more efficient programming language (e.g. C++) are all options that we will investigate to speed up computations and be able to perform our reconstruction inline at the scanner.

One of the main limitations lies in the number of clusters chosen for the reconstruction and in the criterion used for their selection. Currently, we used the size of the clusters, i.e., the amount of data used for the gridded image reconstruction, to select the four most populated clusters. These more populated clusters result in images with higher SNR and consequently guarantee a good performance of the registration algorithm. Moreover, selecting the four most populated clusters allowed us to have uniform reconstruction parameters and comparable reconstruction times for all subjects. Having considered these arguments, we could argue that choosing a fixed number of four clusters may not be optimal for all cases. The SIMBA clustering allows in a very fast and data-driven way to select a resting phase of the heart, while at the same time discarding bulk movement. In the future, we will consider the implementation of a dynamic and automated way to select the clusters, by computing an image quality metric combined with the evaluation of the quality of the registration to have an individually optimized number of clusters.

With respect to the image quality metric, we chose an automated score assignment to evaluate the relative increase or decrease in image quality between the different reconstruction techniques. Piccini et al. claim that this algorithm can differentiate image quality of the same dataset reconstructed with different techniques [32]. In our case, it allowed for an automated, fast, and unbiased evaluation of all 3D images to compare different reconstruction techniques. We observed an increase in scores in 18 out of 24 cases with XD-MC-SIMBA, while image quality seemed equally improved in the other 6 cases by visual inspection. For 5 out of these 6 cases the scores differ by only 0.5 or less which is reported to be not perceivable by human observers [32].

One of the advantages of the SIMBA technique is the fact that it does not target a specific physiological phase, hence it is independent of a precise extraction of a clean physiological signal, where specific assumptions on the timings and frequencies of the respiratory and heart rates are imposed. SIMBA overcomes this by directly targeting data similarity, hence moving towards a data selection that is less affected by subject-specific physiological variabilities. In Heerfordt et al. [19] the type of data selected in each cluster was extensively analyzed and also compared to the 5D image reconstruction [14]. It was observed that the most populated cluster preferentially targets a diastolic end-expiratory phase, yet the anatomical sharpness was not adversely affected in rare cases where the algorithm chose end-systolic phases instead. In this work, we considered the improvement of image quality solely from an image reconstruction point of view, without questioning the data selection. When comparing our reconstruction to a resting phase from the 5D free-running reconstruction, we are able to get similar to better image quality at a lower computational expense, especially when looking at fine structures such as the coronary arteries, which may explain the lower assigned image quality scores. However, XD-MC-SIMBA still does not provide us with the functional dynamic information of FRF, which is of high diagnostic impact when considering wall motion abnormalities, for example. In future work we will develop a precise extraction of end-diastolic and end-systolic images, so that we could use this reconstruction framework also for the computation of ejection fractions. We plan to achieve this by improving the dimensionality reduction and clustering steps to have a better understanding of the relationship between these two data analysis steps and the underlying physiology. This reconstruction would be executable in smaller computational times than a 5D dynamic reconstruction [14], and would not require constraints on the amount of data per cluster, as opposed to equally populated bins in the 5D reconstruction [14], minimizing residual intra-cluster motion.

Additionally, each SIMBA cluster contains all the readouts from the selected interleaves. The current SIMBA clustering is thus greatly affected by the temporal sampling of the SI readouts and blurring of cardiac phases could occur if the sampling rate is not high enough compared to the heart rate. A prospective study in which protocols with different sampling frequencies of the SI readouts or with signals acquired at each k-space line could provide more information about the impact of the input on the performance of the SIMBA clustering and consequently on the final image quality.

In this study we focused on ferumoxytol-enhanced free-running CMRA. The use of this iron-based contrast agent enables higher spatial resolution, excellent anatomical definition, and a more accurate evaluation of the origin and course of coronary arteries, even in young patients with CHD [21, 44–46]. In this context, our proposed reconstruction framework minimized the effects of motion artefacts on image quality without making assumptions about the type of motion and its frequency range, resulting in a fully automated and patient-specific technique. This is particularly significant when considering pediatric CHD patients, as irregular respiratory rates and arrhythmia can make the CMR examination non-diagnostic.

This work has limitations. First, the semantic meaning, in terms of cardiac and respiratory phases, of the SIMBA clusters is unknown before image reconstruction, and cannot be controlled. Moreover, even once the images are obtained, we can only guess where these states fall within the respiratory and cardiac cycles. By not having control over this, the motion between neighboring clusters might be challenging to compensate for. Furthermore, in this work we only consider the largest cluster, which may not yield the ideal anatomical state to visualize the coronary arteries. Finally, our technique is still addressing the reconstruction of data with quite low undersampling factors (below 5.4), which leaves the unexplored potential to address the reconstruction of datasets with much higher undersampling factors, and resultant potential scan time reduction.

Future work should focus on studying the performance of our proposed technique in patients with highly irregular breathing and heartbeat patterns. Additionally, we should also apply it for the reconstruction of datasets acquired with slow infusion of gadolinium [47], and extend our proposed technique to non-contrast CMRA, such as bSSFP with native contrast.

## Conclusion

We successfully implemented a new reconstruction framework (XD-MC-SIMBA) for the effective suppression of respiratory and cardiac motion artifacts in free-running acquisitions. This technique improves data efficiency compared to the original SIMBA method without requiring a reordering of the data according to physiological cycles, which is a novel finding compared to other published motion-resolved reconstructions. We showed that SIMBA clusters can be considered as a new dynamic dimension in a CS reconstruction, exploiting the redundant information. Moreover, we maximized sparsity in the clustering dimension by adding an inter-cluster motion compensation. Compared to the original approach, XD-MC-SIMBA resulted in a significantly improved image quality and coronary artery visibility of ferumoxytol-enhanced cardiac images in patients with CHD. There was no significant difference in image quality metrics between XD-MC-SIMBA and FRF, with XD-MC-SIMBA allowing for a better visualization of finer anatomical features and lower computational times. Future work will aim to further validate this technique in non-contrast-enhanced whole-heart free-running CMRA.

## Supporting information

S1 Fig Reconstruction results for XD-SIMBA and XD-MC-SIMBA, for four regularization parameters λ. For λ = 0.003 and λ = 0.03 we do not perceive any significant difference in image quality or image sharpness by comparing images of the same reconstruction (XD-SIMBA or XD-MC-SIMBA) or images of different reconstructions (XD-SIMBA vs. XD-MC-SIMBA). Moreover, for these two λ values we do not see big improvements compared to the original SIMBA either. However, for λ = 0.3, the final value chosen in our work, we have a significant reduction in noise for XD-MC-SIMBA compared to both SIMBA and XD-SIMBA, without compromising on sharpness or image conspicuity (e.g. the valve leaflets are better visible in XD-MC-SIMBA). Conversely, for XD-SIMBA several features (e.g. the liver dome and the papillary muscles) are blurrier for λ = 0.3, which means that for XD-SIMBA we should use λ = 0.03. Finally, a too high regularization term λ = 3 blurs the cardiac anatomy, in both XD-SIMBA and XD-MC-SIMBA and creates an overly regularized image in XD-MC-SIMBA.
(DOCX)

S2 Fig Analysis of all cluster sizes and shapes for all subjects. To be noted that subjects have different number of clusters. We report the following: N = 10 for 1 subject, N = 11 for 2 subjects, N = 12 for 1 subject, N = 13 for 5 subjects and N = 14 for the remaining 15 subjects. A. The size, corresponding to the number of readouts, of each cluster, ordered by largest to smallest. B. The sparsity, which is equal to the mean of the within-cluster point to point distances. The higher this value the more sparsely distributed the data in the cluster. C. The uniformity of the data in k-space, calculated as the distance between readouts and their four closest neighbors, on a unit sphere. We can observe how going down with the cluster’s size, the sparsity increases, meaning that the data in the clusters is more sparsely distributed. However, the very similar values of uniformity in k-space indicate that the factor contributing to this sparsity in the clusters is motion- and not trajectory-dependent artefacts. This result is in line with our hypothesis that the largest cluster targets more precisely a resting phase of the heart, while the smaller the cluster the more data in slightly different anatomical configurations is clustered together.
(DOCX)

S3 Fig Examples of image registration with NiftyReg. A. Gridded images from one SIMBA cluster: the reference and moving images are very similar, with a slight change in the respiratory liver position (as visible in the difference image). This small deformation is completely corrected after image registration using NiftyReg, as visible in the image difference in which only residual noise is present. The magnitude of the computed deformation field is also shown as a colormap, highlighting the highest deformation at the level of the lung-liver interface. B. Example of gridded images from another SIMBA cluster in which the reference and moving images are in very different cardiac phases. After image registration we are able to correct for these large differences. The residual uncorrected features, as visible in the image difference, are mostly due to differences in contrast (e.g. blood flow dephasing artefacts in systolic phases). The magnitude of the deformation field shows the highest deformation at the level of the heart, mostly at the location of the pulmonary artery and the left ventricle.
(DOCX)

S4 Fig Summary of all image quality metrics for each figure in the paper. (DOCX)

S1 Appendix Description of the deep neural network used for the automated assignment of image quality scores (IQS). The algorithm is the one published by Piccini et al., consisting of a deep convolutional neural network (A) trained to reproduce the grading performance of an expert observer. This image quality assessment algorithm (IQ-DCNN) was trained, optimized and cross-validated on a database of 324 3D whole-heart cardiac MRI scans. The final architecture was tested on 100 scans. All scans were performed on a 1.5-T clinical MR scanner (MAGNETOM Aera, Siemens Healthcare, Erlangen, Germany) using a research free-breathing and respiratory self-navigated ECG-triggered 3D radial bSSFP sequence. Readers graded each image using a diagnostic quality scale ranging from 0 (poor quality) to 4 (excellent quality), in steps of 0.5 according to the level of artefact, blurring, vessel sharpness and noise. The authors showed that the IQ-DCNN algorithm performed within the range of human intra- and inter-observer agreement. When applied during an iterative compressed sensing reconstruction, it correlated with the cost function at each iteration. Moreover, they showed that the final grade is mostly determined by specific anatomical features in the volume, such as the sharpness of small vessels, and not by general blurriness. These findings motivated the use of the IQ-DCNN algorithm to assess different reconstruction techniques, using compressed sensing, as it proved to be able to identify improvements in image quality.
(DOCX)
