# Supplementary material for: Similarity-driven motion-resolved reconstruction for ferumoxytol-enhanced whole-heart MRI in congenital heart disease
Source: PLoS One. 2024 Jun 13;19(6):e0304612. doi: 10.1371/journal.pone.0304612 (PMC11175540; doi:10.1371/journal.pone.0304612)

**S1 Fig. Reconstruction results for XD-SIMBA and XD-MC-SIMBA, for four regularization parameters λ.** For λ=0.003 and λ=0.03 we do not perceive any significant difference in image quality or image sharpness by comparing images of the same reconstruction (XD-SIMBA or XD-MC-SIMBA) or images of different reconstructions (XD-SIMBA vs. XD-MC-SIMBA). Moreover, for these two λ values we do not see big improvements compared to the original SIMBA either. However, for λ=0.3, the final value chosen in our work, we have a significant reduction in noise for XD-MC-SIMBA compared to both SIMBA and XD-SIMBA, without compromising on sharpness or image conspicuity (e.g. the valve leaflets are better visible in XD-MC-SIMBA). Conversely, for XD-SIMBA several features (e.g. the liver dome and the papillary muscles) are blurrier for λ=0.3, which means that for XD-SIMBA we should use λ=0.03. Finally, a too high regularization term λ=3 blurs the cardiac anatomy, in both XD-SIMBA and XD-MC-SIMBA and creates an overly regularized image in XD-MC-SIMBA.


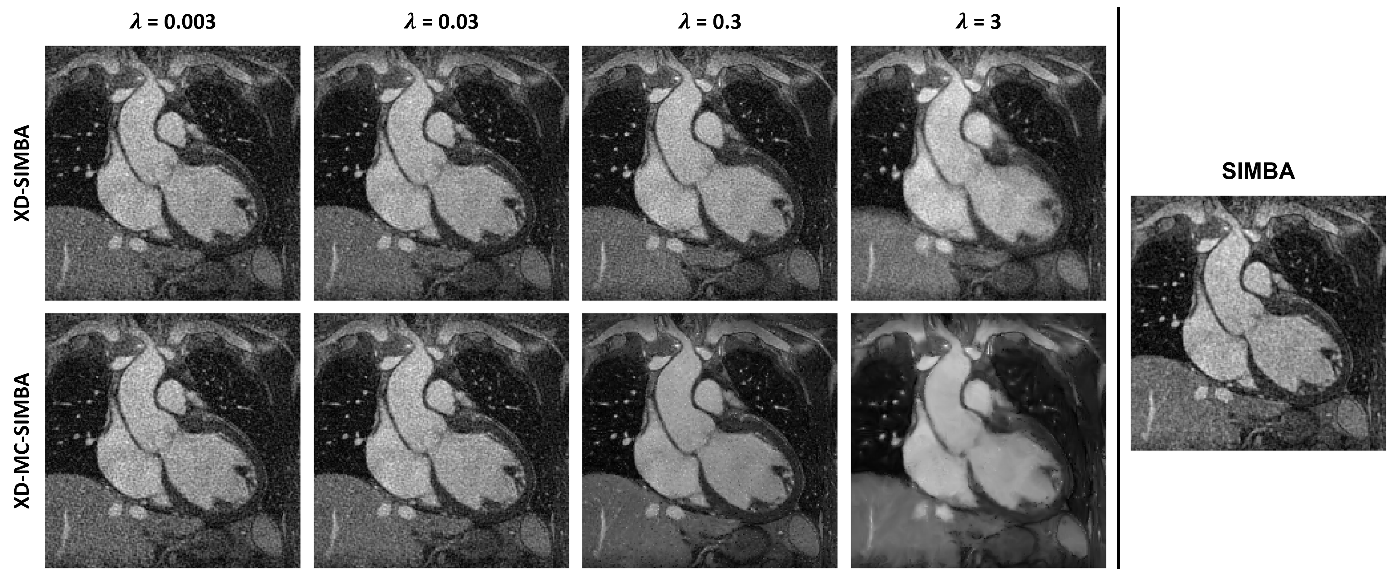

Supplement: S1 Fig — For λ = 0.003 and λ = 0.03 we do not perceive any significant difference in image quality or image sharpness by comparing images of the same reconstruction (XD-SIMBA or XD-MC-SIMBA) or images of different reconstructions (XD-SIMBA vs. XD-MC-SIMBA). Moreover, for these two λ values we do not see big improvements compared to the original SIMBA either. However, for λ = 0.3, the final value chosen in our work, we have a significant reduction in noise for XD-MC-SIMBA compared to both SIMBA and XD-SIMBA, without compromising on sharpness or image conspicuity (e.g. the valve leaflets are better visible in XD-MC-SIMBA). Conversely, for XD-SIMBA several features (e.g. the liver dome and the papillary muscles) are blurrier for λ = 0.3, which means that for XD-SIMBA we should use λ = 0.03. Finally, a too high regularization term λ = 3 blurs the cardiac anatomy, in both XD-SIMBA and XD-MC-SIMBA and creates an overly regularized image in XD-MC-SIMBA. (DOCX) [file pone.0304612.s001.docx]
