# Supplementary material for: Similarity-driven motion-resolved reconstruction for ferumoxytol-enhanced whole-heart MRI in congenital heart disease
Source: PLoS One. 2024 Jun 13;19(6):e0304612. doi: 10.1371/journal.pone.0304612 (PMC11175540; doi:10.1371/journal.pone.0304612)

**S3 Fig. Analysis of all cluster sizes and shapes for all subjects.** To be noted that subjects have different number of clusters. We report the following: N=10 for 1 subject, N=11 for 2 subjects, N=12 for 1 subject, N=13 for 5 subjects and N=14 for the remaining 15 subjects. **A.** The size, corresponding to the number of readouts, of each cluster, ordered by largest to smallest. **B.** The sparsity, which is equal to the mean of the within-cluster point to point distances. The higher this value the more sparsely distributed the data in the cluster. **C.** The uniformity of the data in k-space, calculated as the distance between readouts and their four closest neighbors, on a unit sphere. We can observe how going down with the cluster’s size, the sparsity increases, meaning that the data in the clusters is more sparsely distributed. However, the very similar values of uniformity in k-space indicate that the factor contributing to this sparsity in the clusters is motion- and not trajectory-dependent artefacts. This result is in line with our hypothesis that the largest cluster targets more precisely a resting phase of the heart, while the smaller the cluster the more data in slightly different anatomical configurations is clustered together.


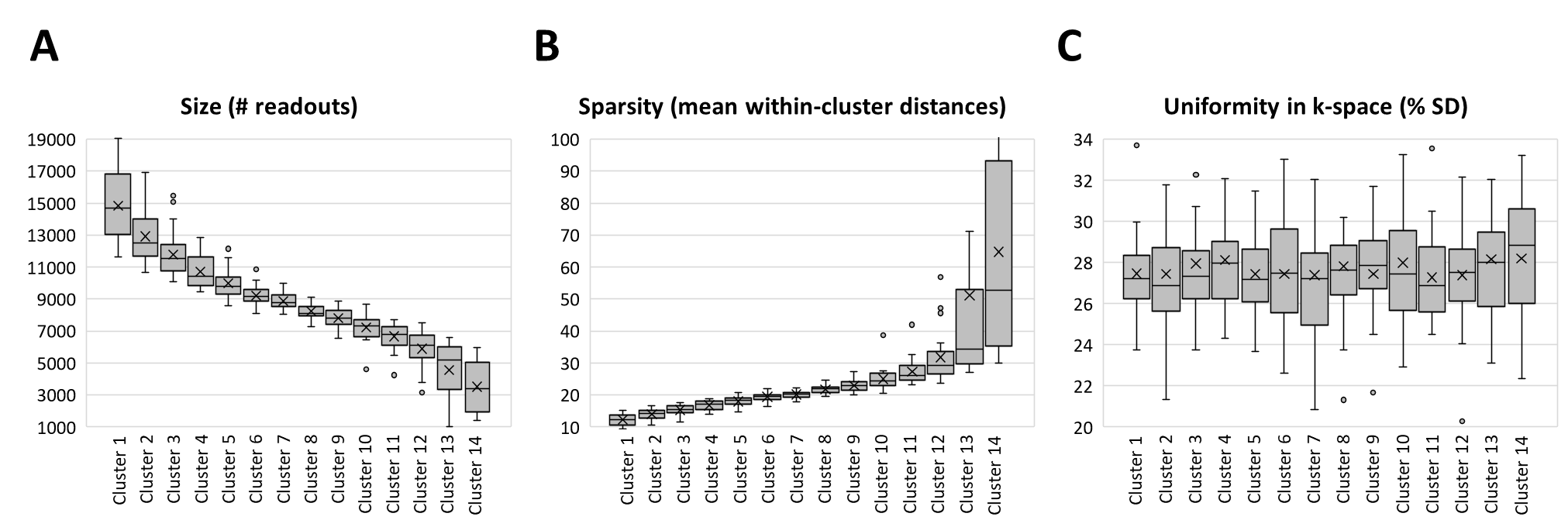

Supplement: S2 Fig — To be noted that subjects have different number of clusters. We report the following: N = 10 for 1 subject, N = 11 for 2 subjects, N = 12 for 1 subject, N = 13 for 5 subjects and N = 14 for the remaining 15 subjects. A. The size, corresponding to the number of readouts, of each cluster, ordered by largest to smallest. B. The sparsity, which is equal to the mean of the within-cluster point to point distances. The higher this value the more sparsely distributed the data in the cluster. C. The uniformity of the data in k-space, calculated as the distance between readouts and their four closest neighbors, on a unit sphere. We can observe how going down with the cluster’s size, the sparsity increases, meaning that the data in the clusters is more sparsely distributed. However, the very similar values of uniformity in k-space indicate that the factor contributing to this sparsity in the clusters is motion- and not trajectory-dependent artefacts. This result is in line with our hypothesis that the largest cluster targets more precisely a resting phase of the heart, while the smaller the cluster the more data in slightly different anatomical configurations is clustered together. (DOCX) [file pone.0304612.s002.docx]
