# Supplementary material for: Similarity-driven motion-resolved reconstruction for ferumoxytol-enhanced whole-heart MRI in congenital heart disease
Source: PLoS One. 2024 Jun 13;19(6):e0304612. doi: 10.1371/journal.pone.0304612 (PMC11175540; doi:10.1371/journal.pone.0304612)

**S4 Fig. Examples of image registration with NiftyReg.** A. Gridded images from one SIMBA cluster: the reference and moving images are very similar, with a slight change in the respiratory liver position (as visible in the difference image). This small deformation is completely corrected after image registration using NiftyReg, as visible in the image difference in which only residual noise is present. The magnitude of the computed deformation field is also shown as a colormap, highlighting the highest deformation at the level of the lung-liver interface. B. Example of gridded images from another SIMBA cluster in which the reference and moving images are in very different cardiac phases. After image registration we are able to correct for these large differences. The residual uncorrected features, as visible in the image difference, are mostly due to differences in contrast (e.g. blood flow dephasing artefacts in systolic phases). The magnitude of the deformation field shows the highest deformation at the level of the heart, mostly at the location of the pulmonary artery and the left ventricle.


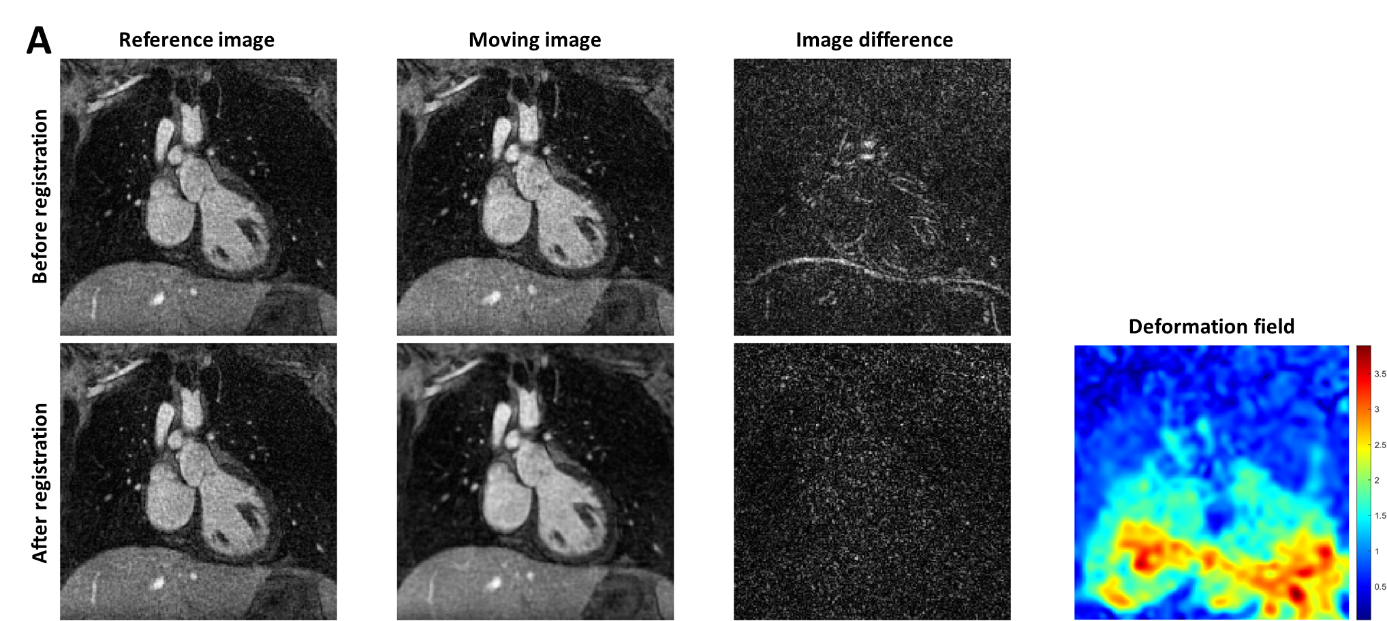


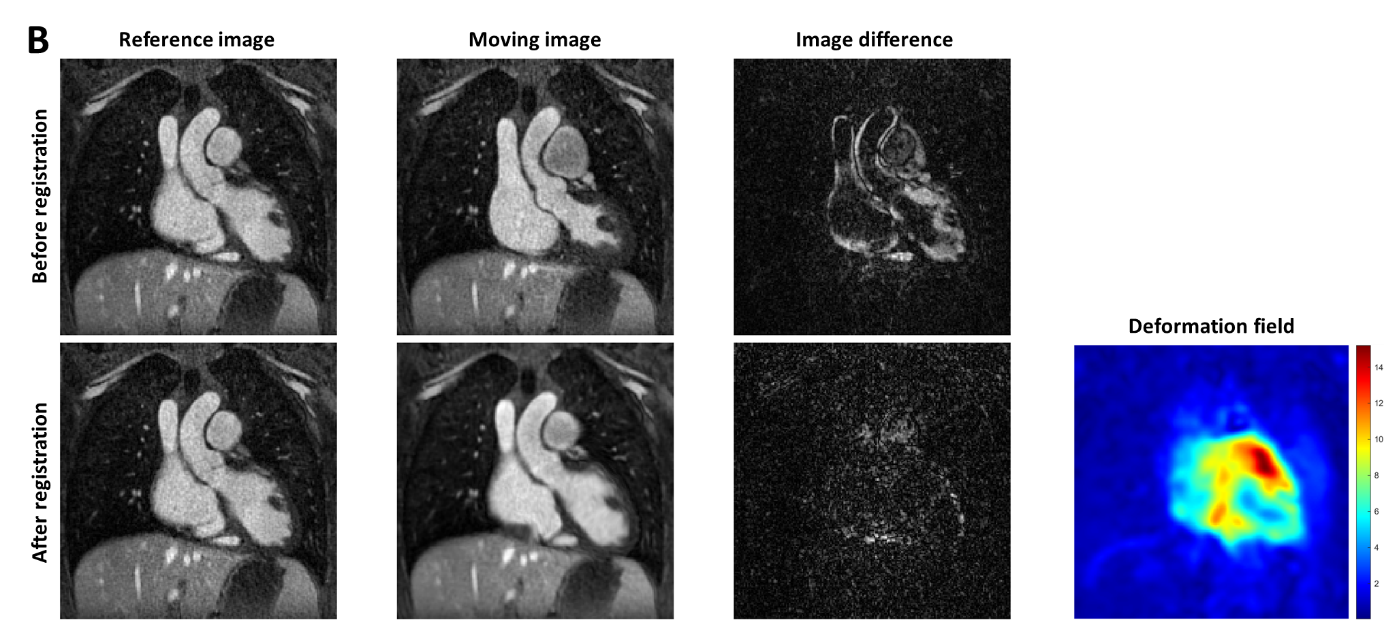

Supplement: S3 Fig — A. Gridded images from one SIMBA cluster: the reference and moving images are very similar, with a slight change in the respiratory liver position (as visible in the difference image). This small deformation is completely corrected after image registration using NiftyReg, as visible in the image difference in which only residual noise is present. The magnitude of the computed deformation field is also shown as a colormap, highlighting the highest deformation at the level of the lung-liver interface. B. Example of gridded images from another SIMBA cluster in which the reference and moving images are in very different cardiac phases. After image registration we are able to correct for these large differences. The residual uncorrected features, as visible in the image difference, are mostly due to differences in contrast (e.g. blood flow dephasing artefacts in systolic phases). The magnitude of the deformation field shows the highest deformation at the level of the heart, mostly at the location of the pulmonary artery and the left ventricle. (DOCX) [file pone.0304612.s003.docx]
