# Supplementary figures and images for: Similarity-driven motion-resolved reconstruction for ferumoxytol-enhanced whole-heart MRI in congenital heart disease
Source: PLoS One. 2024 Jun 13;19(6):e0304612. doi: 10.1371/journal.pone.0304612 (PMC11175540; doi:10.1371/journal.pone.0304612)

**S5 Fig. Summary of all image quality metrics for each figure in the paper.**


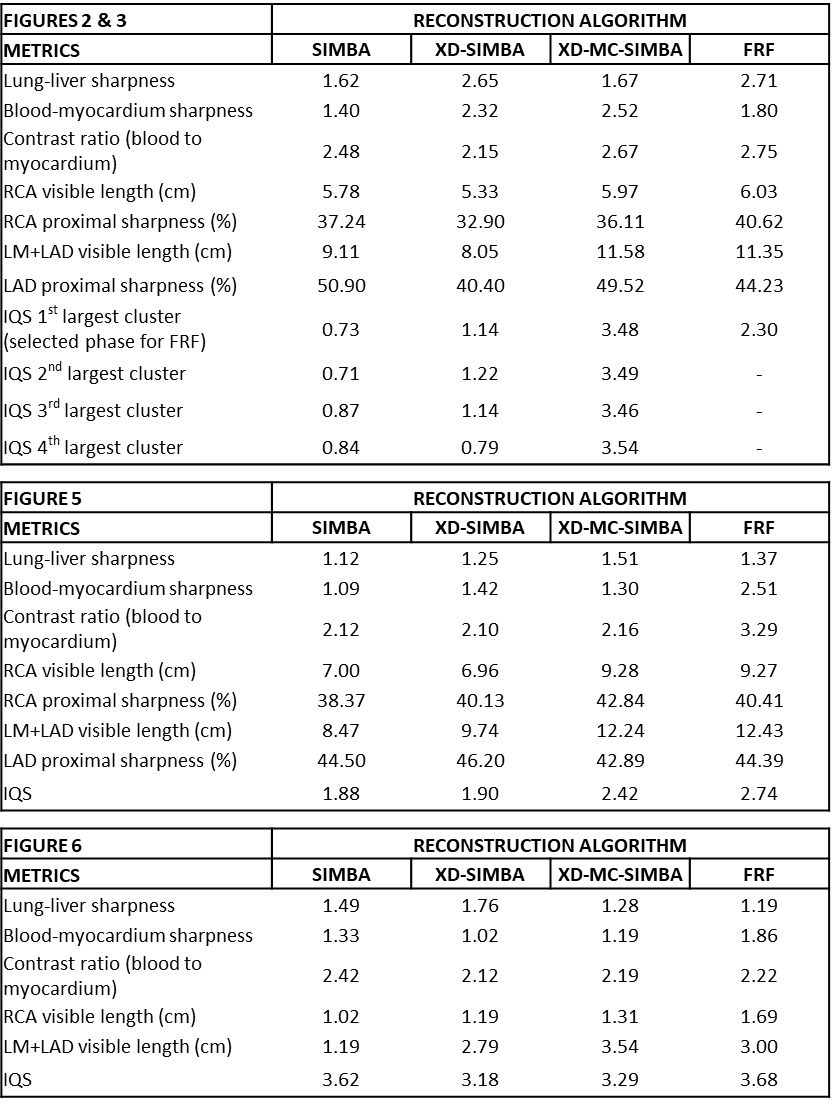

Supplement: S4 Fig — (DOCX) [file pone.0304612.s004.docx]
