# Supplementary material for: Similarity-driven motion-resolved reconstruction for ferumoxytol-enhanced whole-heart MRI in congenital heart disease
Source: PLoS One. 2024 Jun 13;19(6):e0304612. doi: 10.1371/journal.pone.0304612 (PMC11175540; doi:10.1371/journal.pone.0304612)
Supplement: S1 Appendix — The algorithm is the one published by Piccini et al., consisting of a deep convolutional neural network (A) trained to reproduce the grading performance of an expert observer. This image quality assessment algorithm (IQ-DCNN) was trained, optimized and cross-validated on a database of 324 3D whole-heart cardiac MRI scans. The final architecture was tested on 100 scans. All scans were performed on a 1.5-T clinical MR scanner (MAGNETOM Aera, Siemens Healthcare, Erlangen, Germany) using a research free-breathing and respiratory self-navigated ECG-triggered 3D radial bSSFP sequence. Readers graded each image using a diagnostic quality scale ranging from 0 (poor quality) to 4 (excellent quality), in steps of 0.5 according to the level of artefact, blurring, vessel sharpness and noise. The authors showed that the IQ-DCNN algorithm performed within the range of human intra- and inter-observer agreement. When applied during an iterative compressed sensing reconstruction, it correlated with the cost function at each iteration. Moreover, they showed that the final grade is mostly determined by specific anatomical features in the volume, such as the sharpness of small vessels, and not by general blurriness. These findings motivated the use of the IQ-DCNN algorithm to assess different reconstruction techniques, using compressed sensing, as it proved to be able to identify improvements in image quality. (DOCX) [file pone.0304612.s005.docx]

**S2 Appendix. Description of the deep neural network used for the automated assignment of image quality scores (IQS).**

The algorithm is the one published by Piccini et al., consisting of a deep convolutional neural network (A) trained to reproduce the grading performance of an expert observer. This image quality assessment algorithm (IQ-DCNN) was trained, optimized and cross-validated on a database of 324 3D whole-heart cardiac MRI scans. The final architecture was tested on 100 scans. All scans were performed on a 1.5-T clinical MR scanner (MAGNETOM Aera, Siemens Healthcare, Erlangen, Germany) using a research free-breathing and respiratory self-navigated ECG-triggered 3D radial bSSFP sequence. Readers graded each image using a diagnostic quality scale ranging from 0 (poor quality) to 4 (excellent quality), in steps of 0.5 according to the level of artefact, blurring, vessel sharpness and noise. The authors showed that the IQ-DCNN algorithm performed within the range of human intra- and inter-observer agreement. When applied during an iterative compressed sensing reconstruction, it correlated with the cost function at each iteration. Moreover, they showed that the final grade is mostly determined by specific anatomical features in the volume, such as the sharpness of small vessels, and not by general blurriness. These findings motivated the use of the IQ-DCNN algorithm to assess different reconstruction techniques, using compressed sensing, as it proved to be able to identify improvements in image quality.

Reference: D. Piccini et al., “Deep learning to automate reference-free image quality assessment of whole-heart MR images,” Radiol. Artif. Intell., vol. 2, no. 3, pp. e190123–e190123, May 2020, doi: 10.1148/ryai.2020190123.
